# The Story of the Insane from Year to Year

**Published:** 1904-11-26

**Authors:** 


### THE STORY OF THE INSANE FROM YEAR TO YEAR.

Seventeenth Annual Series.

### THE MONTROSE ROYAL ASYLUM.

This asylum contained 679 patients at the beginning of the year and 687 at the end of it. There were 133 first admissions, 28 readmissions, 63 discharged recovered, 11 discharged relieved, and 77 died. The total number under treatment was 840, and the average number resident was 690. The recoveries were 39·12 per cent. of the admissions, and the deaths were 12·22 per cent. of the average number resident. Of the year's deaths, 30 were caused by cerebral and spinal diseases and 14 by consumption. This mortality from phthisis represents 2·02 per cent. of the average number resident, and Dr. Havelock finds that the mortality in the Montrose Asylum from this disease during the previous five years amounted to 15 per thousand of the patients, as compared with a mortality of 20 per thousand in the asylums of Great Britain. Of the 14 cases dying during the year, 9 had the disease on admission. Dr. Havelock isolates these consumptive patients in the receding wings of the asylum, and although he does "not regard this arrangement as an absolutely perfect one, it seems the best that can be devised in the existing buildings"; and he adds that it seems to be "the wisest policy to wait until experience has been gained in the working of these sanatoria" which have been erected at other asylums. The fire appliances were examined during the year by an expert, who reported that the exits were ample, and that the buildings were constructed in such a way as to prevent a conflagration spreading. A stronger fire-pump and enlarged water mains were recommended, so that those who may have friends in the Montrose Asylum may now feel at rest as far as the danger from fire exists.

### CUMBERLAND AND WESTMORLAND ASYLUM.

On the first of January this asylum contained 694 patients and by the 31st of December the number had risen to 724. There were 136 first admissions, 50 readmissions, 65 discharged recovered, 21 discharged relieved, and 64 died. The total number under care during the year was 872, and the average number resident was 707. The recoveries were 34·9 per cent. of the admissions, and the deaths were 9 per cent. of the average number resident. Of the year's deaths 19 were caused by cerebral and spinal diseases, 11 by consumption, and 4 by pneumonia. Of the year's admissions such moral causes as domestic trouble and religion account for the insanity in 26 instances, and among the physical causes hereditary influence is credited with being the cause in 63, previous attacks in 49, drink in 23, and in 39 no cause was ascertained. The asylum is to be enlarged and special provision will be made for the isolation of consumptive patients. Last year the patients increased by 29, and Dr. Farquharson says this increase was "due not so much to a larger number of admissions as to the unfavourable nature of the cases brought to us, resulting in a lowered recovery rate and an accumulation of chronic and hopeless cases." At this asylum, as at others, it has been found "increasingly difficult to draft chronic harmless cases from the asylum to the various workhouses"; and the cause of this difficulty is not far to seek. Referring to criminal patients in county asylums, Dr. Farquharson says they are out of place, and "have a demoralising effect on the other patients." All this has been pointed out for the last 30 years by many medical superintendents, but nothing has been done to remedy the evil, and we hope that Dr. Farquharson and others will hammer away until the Government can be got to listen. The weekly charge for county patients is 9s. 4d. per head. There are upwards of 60 private patients who pay from 14s. to 42s. a week. In our opinion the latter sum is much too high for a county asylum to charge, as it is bringing a rate-supported institution into competition with private asylums.

### PLYMOUTH BOROUGH ASYLUM.

The twelfth annual report of this asylum shows that on January 1st it had 277 patients in residence, and that by the end of the year the number had fallen to 272. There were 50 first admissions, 7 readmissions, 25 discharged recovered, 3 discharged relieved, and 31 died. The total number under treatment was 344, and the average number resident was 270. Calculated on the admissions the recoveries work out at the high percentage of 45·45; and calculated on the average number resident the deaths stand at 11·48, which is also high. Of the year's deaths 17 were caused by cerebral and spinal diseases, 3 by consumption, 2 by other lung diseases, and 2 by colitis. Of the year's admissions it is stated that 13 owed their insanity to domestic troubles, mental anxiety, adverse circumstances, and nervous shock; and among the physical causes, intemperance in drink accounts for 6, hereditary influence for 10, previous attacks for 13, and in 14 no cause was ascertained. The asylum continues full and the additions were not ready for occupation on the date of the report, but the committee say they have used every exertion to expedite the work. In the meantime some chronic patients have been transferred to the workhouse to make room for acute cases. The Devonport Corporation wished to join with the Plymouth Borough in a permanent asylum for both, but the latter authorities would not entertain the proposal; why it is not easy to see as it is beyond doubt that an asylum of say 600 beds can be more economically managed than one of 300 beds, and classification of the patients can be better carried out. The committee record their "appreciation of the zeal and industry" of the medical superintendent and the assistant medical officer. The maintenance rates for borough cases was 10s. 4d., out-borough cases pay 14s., and private patients from 18s. to 25s. per head per week. We look upon the 25s. charge as at least 4s. too high.

